# Influence of *Artocarpus hirsutus* (AH) cellulose micro fiber, bamboo fiber in thermoplastic biocomposites

**DOI:** 10.1038/s41598-025-88058-5

**Published:** 2025-02-07

**Authors:** Sumesh Keerthiveettil Ramakrishnan, Kavimani Vijayananth, Ajithram Arivendan, Muhammad Imam Ammarullah

**Affiliations:** 1 Department of Materials Engineering, Faculty of Mechanical Engineering, Czech Technical University in Prague, Prague 02, 12135, Karlovo Namesti 13 Czech Republic; 2 Department of Mechanical Engineering, Karpagam Academy of Higher Education, Coimbatore, 641021 Tamil Nadu India; 3 Centre for Material Science, Karpagam Academy of Higher Education, Coimbatore, 641021 Tamil Nadu India; 4 Shien Ming Wu School of Intelligent Engineering, South China University of Technology, Guangzhou, 510006 Guangdong China; 5 Department of Mechanical Engineering, Faculty of Engineering, Universitas Diponegoro, Semarang, 50275 Central Java Indonesia; 6 Undip Biomechanics Engineering & Research Centre (UBM-ERC), Universitas Diponegoro, Semarang, 50275 Central Java Indonesia

**Keywords:** Mechanical properties, Natural fibers, Cellulosic filler, Biocomposites, Engineering, Mechanical engineering

## Abstract

In this experiment *Artocarpus hirsutus* (AH) fiber was utilized as the filler material for bamboo fiber (NF)/polyethylene (PE) biocomposites. This was a waste to wealth approach by utilising biomaterial and also can reduce the use of PE plastics. The crystallinity index (Crl) of 45.1%, 56.4%, 67% was observed in AH, alkali treated (NaOH) and cellulose AH fiber respectively. The combination with 20 wt% NF/3 wt% cellulose AH filler observed better tensile and flexural strength. Agglomeration at 4, 5 wt% affects the flexural properties by lesser interfacial adhesion with filler/matrix phase, having properties reducing up to 20.3 MPa. Comparing to cellulose AH filler, both alkali treated and untreated AH filler combinations possess lesser flexural strength. The addition of natural fibers increases the tensile and flexural modulus property with better properties at 30 wt% NF/5 wt% cellulose AH filler combination. The Impact strength doesn’t observe high influence with filler incorporation. This AH fiber hasn’t been explored in detail for mechanical and hydrophilic properties with incorporation with PE matrix. This fabricated composite is suited for bioengineering applications.

## Introduction

Polymer composites are crucial in several sectors because of their remarkable mechanical qualities and resilience. Polyethylene is notable among distinct polymer groups for its high reactivity towards numerous chemical species, making it capable of interacting with a broad range of curing agents^1,2^. Nevertheless, polyethylene thermoplastic resin is subject to several restrictions, such as its lower mechanical qualities when compared to other engineering plastics, its tendency to soften at low temperatures, and its susceptibility to creep under steady stress. These limitations impede its use in high-stress conditions and restrict its longevity in certain high thermal resistance, automotive, biomedical based applications^3,4^.

In order to address these limitations, it is possible to include fillers of different kinds, dimensions, and configurations into polyethylene in order to enhance its characteristics and broaden its range of uses. The effectiveness of reinforcement depends on the physical and chemical properties of the filler, including its kind, amount, distribution, and interaction with the polymer matrix^5,6^. Fillers may be classified into two main categories: inorganic metal oxides (such as zinc, iron, copper, and aluminium) and organic fillers (including fullerenes, carbon black, carbon nanotubes, graphene, and their derivatives). These additives have been widely used to improve the mechanical characteristics of polymer composites, particularly epoxy resin, consequently increasing tensile strength and fracture toughness^7–9^.

Nevertheless, the apprehensions around synthetic fillers, such as their exorbitant price, lack of biodegradability, and difficulties in recycling, have generated a need for bio-based substitutes. Switching from synthetic to natural fillers, such as cellulose fibers, bamboo fibers provides a multitude of benefits^10–12^. The natural fibers mentioned, such as coir, sisal, kenaf, banana, bamboo, hemp, pineapple leaf fiber, and jute, possess characteristics of being lightweight, relatively strong, and environmentally benign to design a biocomposites. They mostly function as major reinforcements in composites because of their abundant cellulose content, whereas fillers act as secondary reinforcements to cover gaps or meet special needs^13,14^. Recently, there has been a significant trend towards using advanced bio-based particles such as biochar, lignin, cellulose, and chitosan to improve the characteristics of composites. Cellulose has attracted interest because to its capacity to enhance the stiffness, resilience, and durability of fibers and composites, while also promoting biomaterial and biocomposites application^15–17^.

Research investigations have emphasized the strengthening impact of microcrystalline cellulose (MCC) and cellulose nanocrystals (CNCs) in improving the mechanical characteristics of polymers. Cellulose whiskers, derived from several sources including wood, natural fibers, fungus, and algae, has several benefits such as a high aspect ratio, low density, cost-effectiveness, low toxicity, exceptional thermal and mechanical capabilities, biocompatibility, and biodegradability, makes a suitable candidate for automobile, biomedical applications^18–20^. Although progress has been made in using natural fibers and cellulose-based fillers, there is still unexplored potential in investigating other sources of cellulose^21–23^. Agricultural leftovers such as banana fruit peduncles, coffee husks, office waste papers, and throwaway cups may be used as effective renewable sources for the production of cellulose derivatives. Transforming these lignocellulosic materials into products with increased value not only has positive effects on the environment but also reduces the strain on solid waste management. Chemical procedures like as alkaline treatment and bleaching are used to pretreat lignocellulosic resources, making it easier to extract cellulose. This enables the creation of biocomposite materials that are sustainable and ecologically benign^24–26^.

*Artocarpus hirsutus*, an extraordinary plant species indigenous to India and diverse tropical areas, has considerable potential as a renewable resource. India has a large population of *Artocarpus hirsutus* plants, with a significant number of them being found in Tamil Nadu. This plant produces edible fruits that are highly valued worldwide for their nutritional content and affordability, making them essential ingredients in many different types of cuisine. Moreover, compounds derived from *Artocarpus hirsutus* have significant importance in culinary applications, namely in the creation of desserts, confections, and drinks. In addition, the plant provides rich fibers obtained from its leaf sheaths and fruits, which are used in the production of ropes, high-quality fabrics, baskets, and other handicrafts^27^. Nevertheless, the wasted fruit peduncles of *Artocarpus hirsutus* are often considered agricultural waste, resulting in environmental concerns such as land use problems and garbage buildup. Transforming this biomass into fibrous reinforcing elements for polymer matrices offers a chance to enhance its worth and address environmental issues. Utilizing the cellulosic biomass from *Artocarpus hirsutus* may help reduce waste volume and contribute to the creation of lightweight biocomposites for structural and biomedical applications, therefore alleviating the pressure on solid waste management. The plentiful presence of *Artocarpus hirsutus* fruit peduncles, which contain a high amount of lignocellulosic fibers, enhances the pace of production of strengthening materials, so contributing to the growing need for sustainable manufacturing solutions worldwide. Utilizing the capabilities of *Artocarpus hirsutus* and its by-products may promote sustainability, optimize resource use, and drive innovation in several industries, all while effectively tackling environmental issues^28,29^.

This study aims to emphasize the significance of investigating unexplored sources of cellulose in order to promote the production of environmentally friendly biocomposite materials and contribute to efforts in environmental conservation. This work used *Artocarpus hirsutus* (AH) seed scale fibers of three types, bamboo natural fibers (NF) for fabricating ecofriendly polyethylene (PE) polymer composites. These composites were taken for tensile, flexural and impact testing along with tensile and flexural modulus properties. Water absorption properties, void, hardness test followed by TGA (Thermogravimetric analysis) and differential scanning calorimetry (DSC) testing was also considered. This also could improve the applications of natural fiber-based polyethylene composites and to reduce usage of polyethylene polymer plastic. This AH fiber hasn’t been explored in detail for mechanical, thermal and hydrophilic properties with incorporation of PE matrix.

## Materials and methods

### Materials

**Fig. 1:**
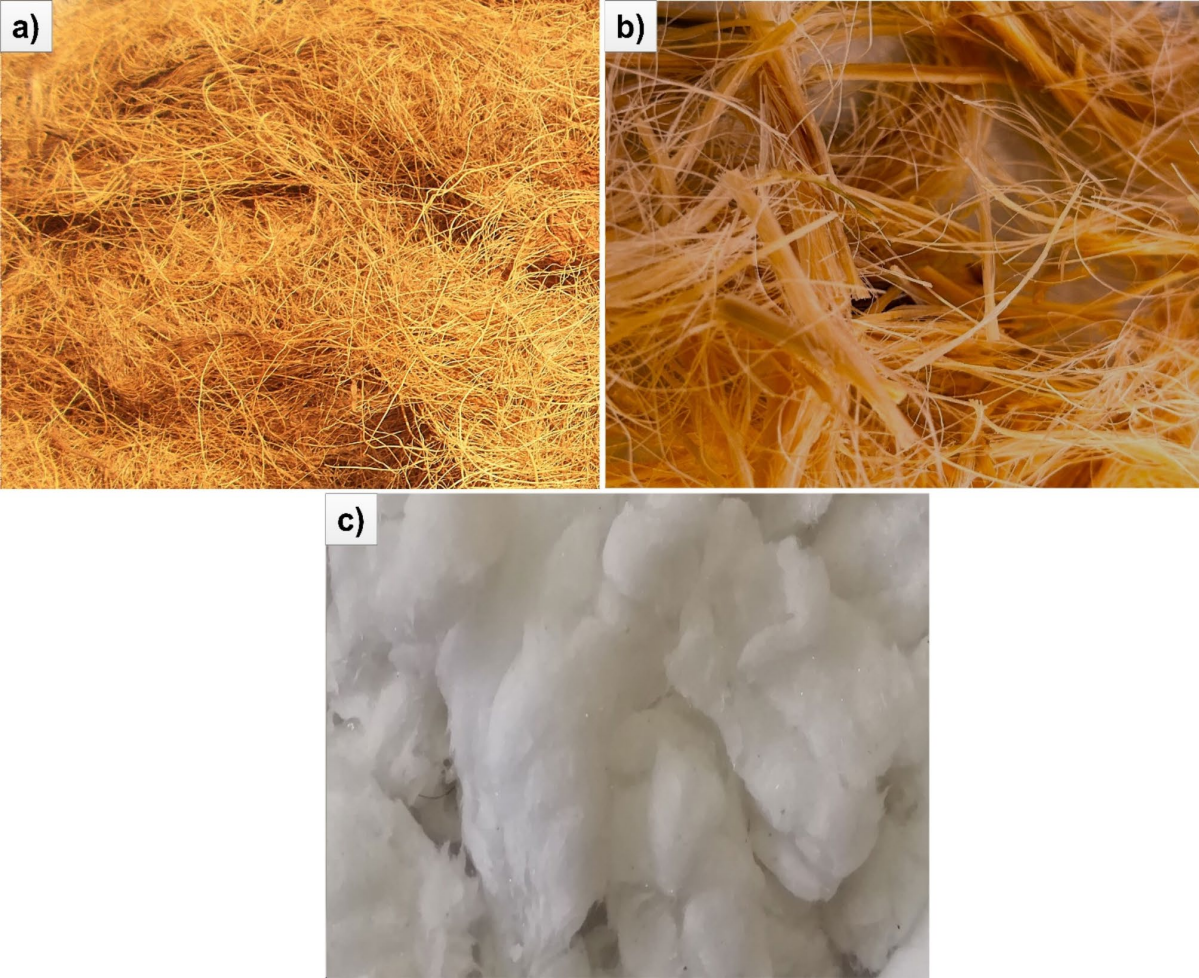
*Artocarpus hirsutus* (AH) fibers. (**a**) AH fiber (untreated), (**b**) Fiber after alkali treatment, (**c**) Cellulose fiber after bleaching process.

The Bamboo fibers (NF) gathered from fiber institute, Chennai, Tamilnadu, India was used as the reinforcement. The *Artocarpus hirsutus* (AH), alkali treated AH and cellulose AH fibers were extracted from the plants grown in Thrissur, Kerala, India was used as fillers (Fig. 1). The similar procedure was followed for the extraction process^24,30^. This natural reinforcement, fillers were incorporated with linear low-density polyethylene (PE) (E24065) taken from Reliance industries limited India. The properties of cellulose AH fibers were showed in Table 1, having richness in cellulose content with 81.6% followed by lesser hemicellulose and lignin content with 9.1%, 8.4% respectively. It has higher tensile strength of 213.2 MPa in comparison to bamboo fibers with lesser than that.

**Table 1:** Properties of fibers used.

Properties	Units	AH fiber (Raw Fiber)	Alkali treated AH Fiber	Cellulose AH fiber	Bamboo fiber	Bamboo fiber^9^	Alkali treated Bamboo fiber^10^
Cellulose	wt%	52	63	81.6	54.3	52.8	63.9
Hemicellulose	wt%	27.1	21.1	9.1	24.4	25.2	19.35
Lignin	wt%	19.8	14.9	8.4	19.2	19.9	14.9
Wax	wt%	-	-	-	0.21	0.16	0.13
Ash	wt%	0.81	0.71	0.6	1.51	1.39	1.26
Density	Kg/m^3^	1413	1415	1421	843	855	857
Tensile Strength	MPa	162.4	184.6	213.2	173	183	213
Youngs Modulus	GPa	7.2	7.4	7.5	7.21	7.16	7.24
Diameter	µm	145–163	121–129	92–97	140–220	120–200	100–170

### Fabrication of composite samples

**Fig. 2:**
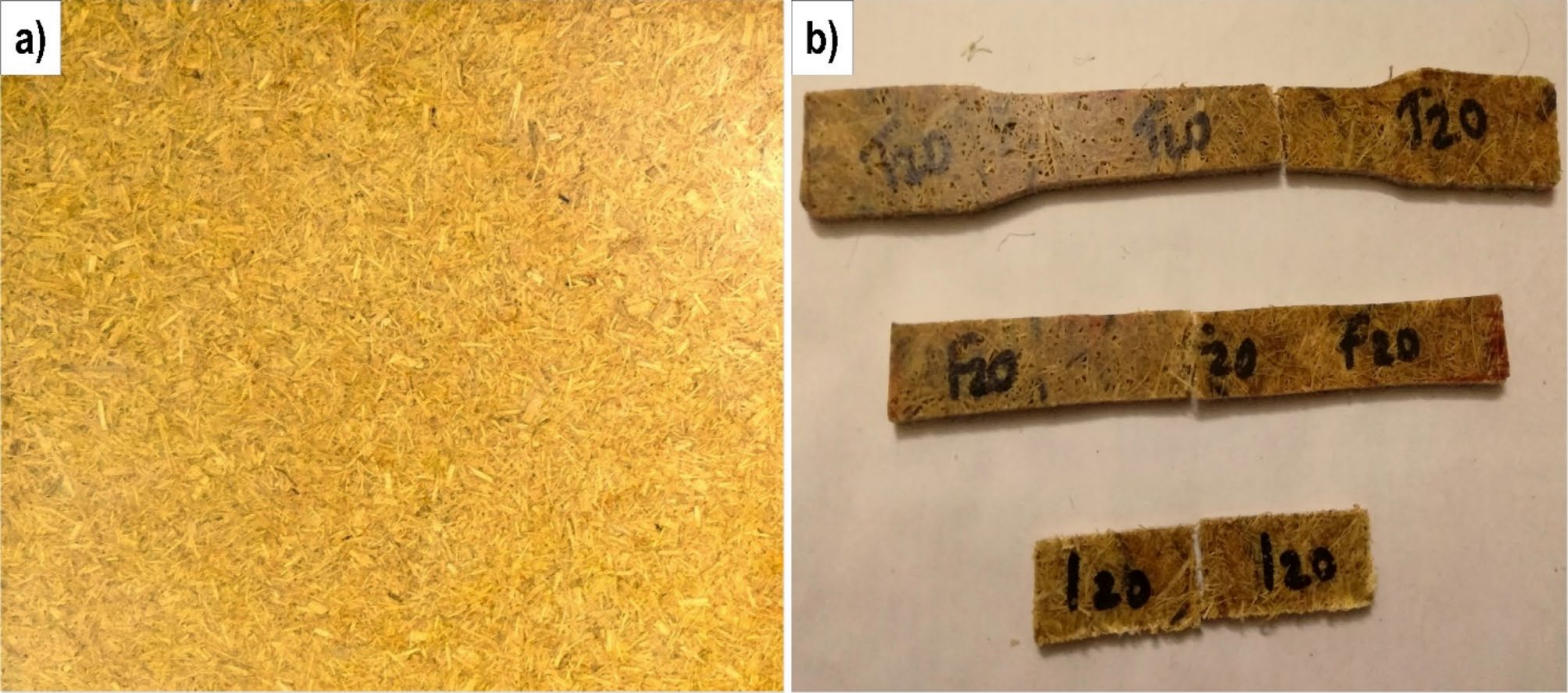
Prepared composite samples. (**a**) Samples after compression moulding, (**b**) samples cropped for mechanical testing.

The bamboo natural fibers (NF) were used at 10, 20 and 30 wt% cropped with a fiber length of 1 cm. In this research, AH fibers were considered as fillers for enhancing the properties of bamboo/PE combination. Three types of *Artocarpus hirsutus* (AH) fillers were utilized here, initial one is the untreated AH fiber. Second filler is AH fiber after alkali treatment process (17.5 wt% NaOH treatment) and the third one is the Cellulose AH fiber after the bleaching process. In order to fabricate the polymer composite sample using natural fibers and *Artocarpus hirsutus* (AH) filler, extrusion processing followed by compression moulding process was utilized. In the single screw extrusion process four zones were there having Zone 1 with 190 °C which is near to nozzle, Zone 2, 3 with 200 °C and Zone 4 with 190 °C near to hooper of the single screw extruder. Speed of the screw was fixed at 6 rpm. The final composite strip coming outside the extruder was shredded using shredding machine and these pellets were used in the compression moulding fabrication with 200 °C at 14 MPa. The prepared sample was showed in Fig. 2.

### Material characterization

Tensile testing was undertaken using Universal testing machine (UTM) (Tinius Olsen H10KL) with ASTM D628 standard (a dumbbell shaped) with gauge length and crosshead speed of 50 mm, 50 mm/min for the composite specimen. Flexural testing was carried out using ASTM D790 a rectangular shaped sample with UTM machine having three-point testing set up. Impact test was carried out using Izod impact test machine CEAST 7.5J (Instron, USA) using ASTM D256 standard notched sample with 2 mm offset. The SEM testing (QUANTA 200 model) was done using cryogenic samples by dipping in liquid nitrogen for making brittle and breaking the sample to find the morphological study. The brocken samples were coated with chromium for proper passing of electrons through it. The distribution of fillers in PE resin, the adhesion of PE matrix/filler/fiber phase is also detected here. Water absorption property was carried out for 31 days (ASTM D570) with samples kept in distilled water (1). Water absorption was checked with change in weight after dipping in distilled water for every single day. Three samples from one combination were checked with square shaped sample of 25 × 25 mm dimension.

1 $${\text{Water}}\;{\text{absorption}}\;{\text{rate }}\left( {{\text{WA}}} \right) \, = \frac{{(W_{I} - W_{O} )}}{{W_{O} }} \times 100$$

W_O,_ W_I_ were the sample weight (g) before and after dipping in distilled water.

The functional groups of *Artocarpus hirsutus* (AH), alkali treated (NaOH), cellulose AH fiber was tested using FTIR testing for detecting presence of cellulose, hemicellulose and lignin in it. The FTIR testing (NEXUS 6800-50 Model) was used with spectral resolution of 2 cm^−1^ from 400 to 4000 cm^−1^. The XRD testing (X Pert PRO, 40 kV, 20 mA) was carried out for finding peaks, crystallinity index, crystallite size of the AH fillers after different treatments with results from 5° to 60° angle range. The crystallinity index (Crl) (2) and crystallite size (CrS) (3) was calculated using Segal empirical method and Scherer’s equation.

2 $$Crl=\frac{{I}_{200}-{I}_{am}}{{I}_{200}}\times 100$$

In that ${I}_{200}$ is the highest peak intensity at 2 0 0 lattice plane with 21.6° as the 2θ value, here ${\text{I}}_{\text{am}}$ is low intensity at 18.4–19° as 2θ value which is the amorphous area. Crystalline size (CrS) equation is showed below.

3 $$CrS= \frac{0.89\lambda }{\beta \text{cos}\theta }$$

In this, ‘$\lambda$’ denotes X ray wavelength constant with 0.154 nm, ‘$\beta$’ called as Full width at half maximum, ${\uptheta }$ denotes the Bragg angle.

## Results and discussion

### XRD, FTIR, SEM testing

**Fig. 3:**
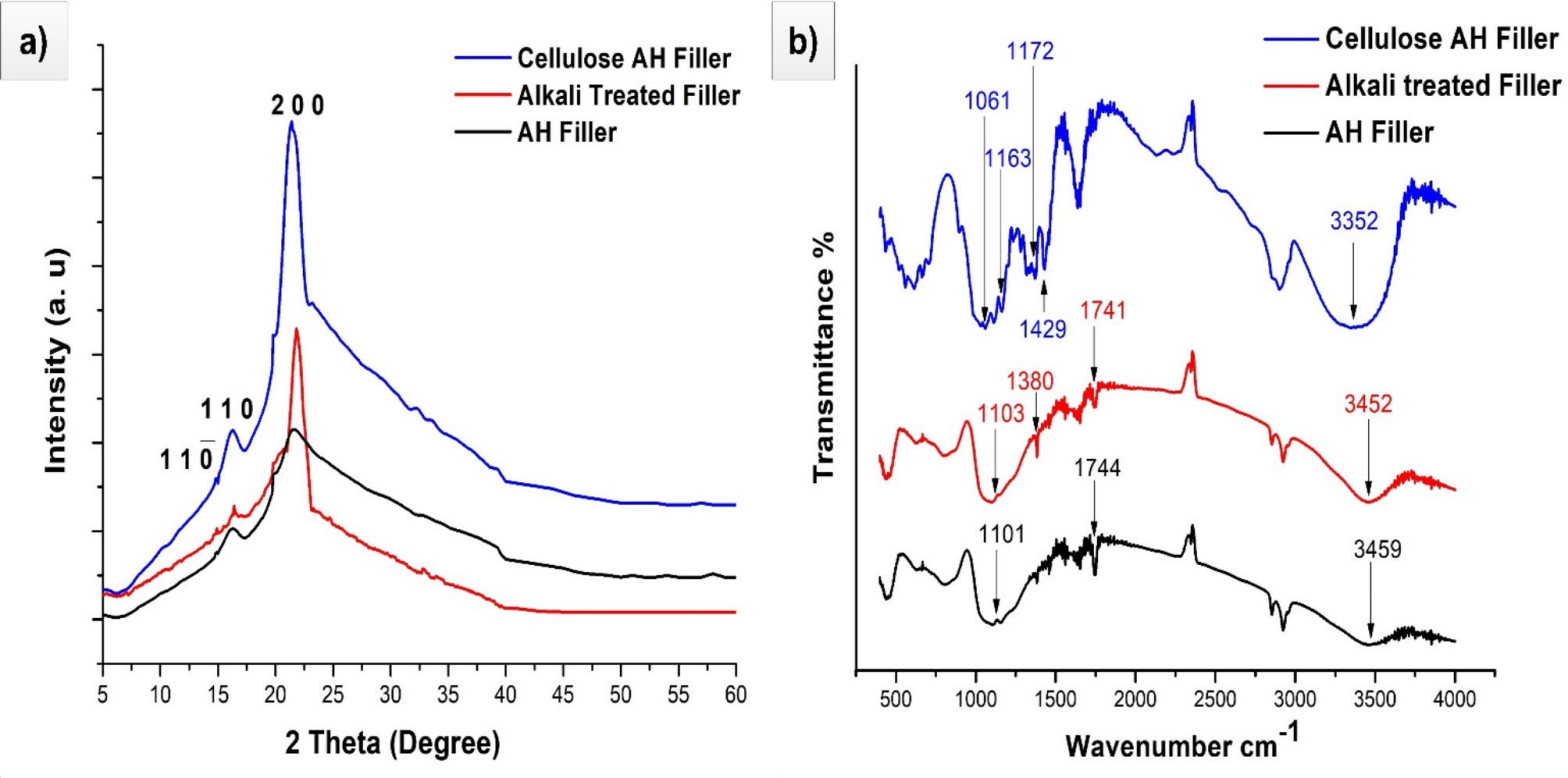
(**a**) XRD and (**b**) FTIR results of *Artocarpus hirsutus* (AH) filler at different surface treatments.

The peak seen at a wavelength of 16.3^o^ corresponds to crystallographic plane at 110 (Fig. 3a). On the other hand, the narrower peak observed at a wavelength of 21.6° is connected with the crystallographic plane at 200^12^. It showed no transformation in its structure with any of the filler using various surface treatments. The crystallinity index (Crl) of 45.1%, 56.4%, 67% was showed in *Artocarpus hirsutus* (AH) filler, alkali treated (NaOH) filler, cellulose AH filler respectively. Crystallinity index in cellulose AH filler was higher due to richness in cellulose than hemicellulose lignin and different other content. This point out the effectiveness in different acid treatments with AH filler. The crystallite size (CrS) of 4.8 nm (Cellulose AH filler), 2.8 nm (alkali treated AH filler), 2.2 nm (AH filler) was observed in different fillers. Higher CrS fillers have the capability for better bonding with polymer matrix composites^7^.

Following the application of an alkaline and different acid treatments, there is a noticeable amplification in intensity of filler peaks. Moreover, the celluloses obtained from the treated filler have significantly elevated peak intensities. The improvement may be described to the chemical alteration of the filler and its surface chemistry during the treatments^13^. Modifying the substance improves functional groups, leading to stronger intensity peaks when analysing it. Furthermore, the treatment stimulates the surface of the filler, resulting in the formation of additional reactive sites that enhance interactions. In addition, residues resulting from the treatment procedure may indirectly contribute to the observed amplification of intensity peaks. Moreover, the treatment results in the creation of rougher surfaces, which in turn provide a greater number of locations for interaction.

**Fig. 4:**
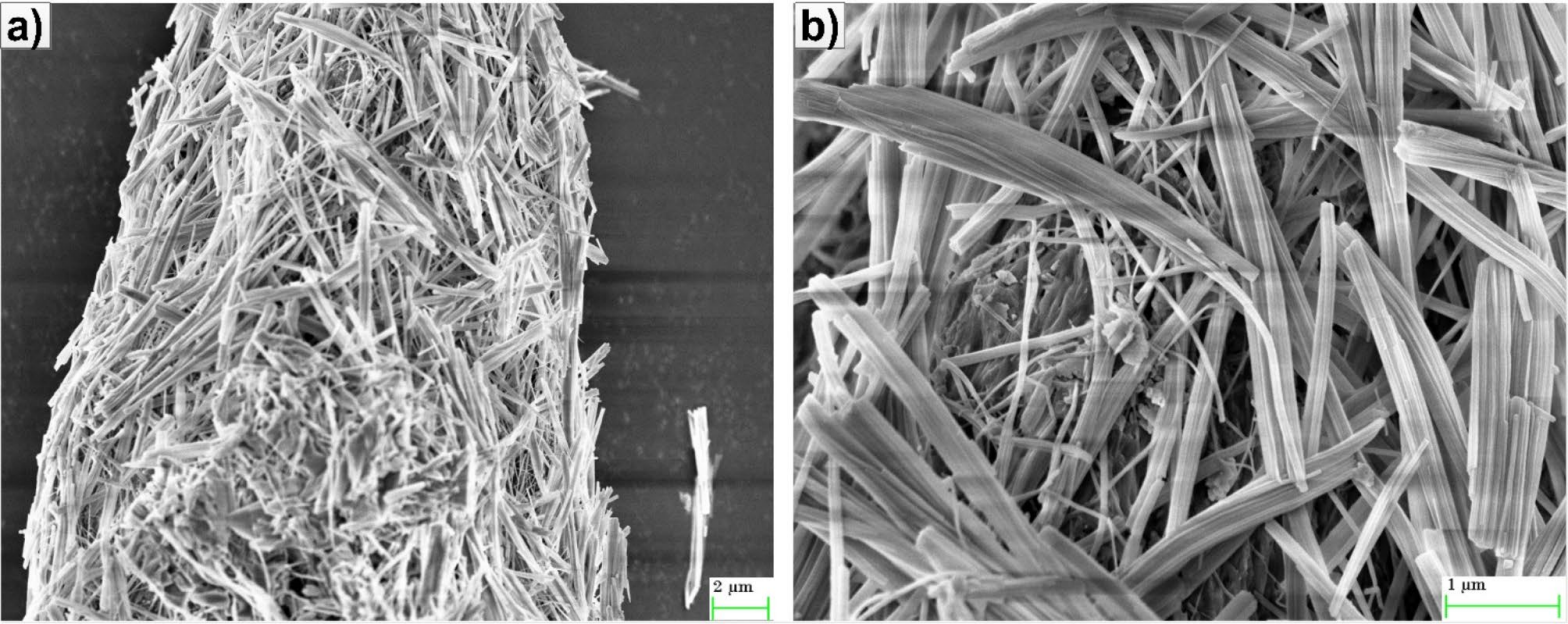
SEM image of cellulose *Artocarpus hirsutus* (AH) filler.

In FTIR (Fig. 3b), the presence of a peak at 1101 cm^−1^ indicates the stretching vibration of a C–O group in the untreated filler material. However, following alkaline treatment, this peak shifts to 1103 cm^−1^, suggesting a modification in bonding of C–C functional group. The presence of minor peaks at 1061 cm^−1^ and 1163 cm^−1^, which are indicative of cellulose, can be observed. These peaks correspond to specific vibrational modes of cellulose molecules, with the peak at 1061 cm^−1^ typically associated with the stretching vibration of C–O–C bonds, and the peak at 1163 cm^−1^ associated with stretching vibration of C–O bonds in cellulose^28^. The primary peak and the existence of secondary peaks linked to cellulose indicate alterations caused by alkaline treatment, presumably including chemical modifications or structural changes. Furthermore, the peak seen at 1744 cm^−1^ may be attributed to stretching vibration of the carbonyl (C=O) group. This suggests the presence of ester groups containing carbonyl in the raw material. The peak at 1741 cm^−1^ after alkaline treatment indicates a modification in the bonding of the carbonyl group as a result of treatment. The peak seen at around 1163 cm^−1^ in the cellulose spectrum corresponds to the asymmetric stretching vibration of the C–O–C bond inside the cellulose molecule, thereby confirming existence of cellulose. Moreover, the peak seen at 3459 cm^−1^ is often attributed to the stretching vibration of O–H bonds, which is frequently linked with hydroxyl groups, such as those present in water molecules. The observed peak to 3452 cm^−1^ after the treatment indicates a potential alteration in the hydroxyl group concentration resulting from the alkaline treatment^29,31^. A fiber like structure is clearly visible with the SEM image of cellulose AH filler (Fig. 4).

### Tensile testing

**Fig. 5:**
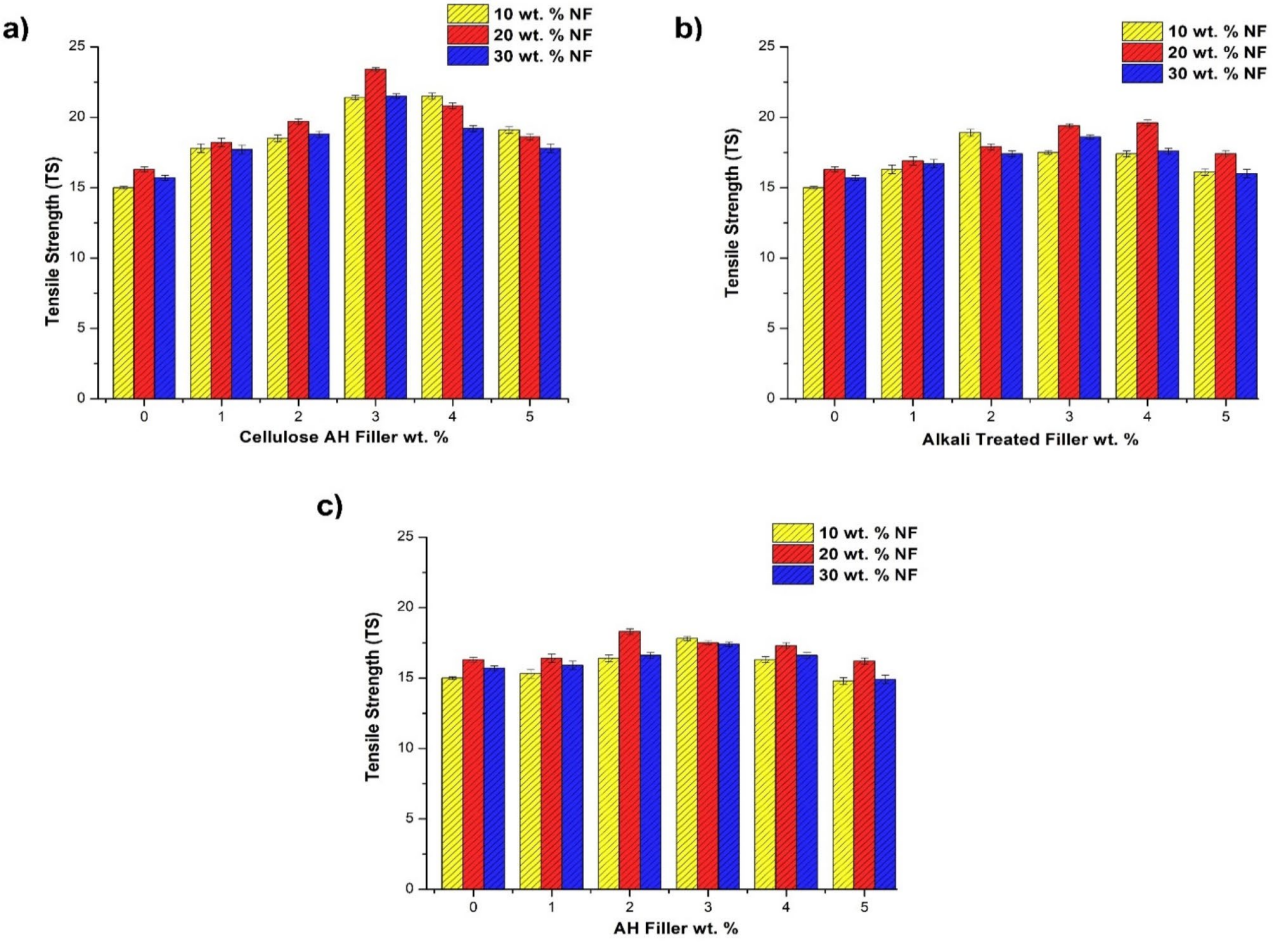
Tensile strength (TS) of bamboo fiber (NF)/*Artocarpus hirsutus* (AH) filler combinations. (**a**) cellulose AH filler, (**b**) Alkali treated filler and (**c**) untreated AH filler

In the initial step, 10, 20 and 30 wt% bamboo natural fibers (NF) were incorporated with 0–5 wt% of *Artocarpus hirsutus* (AH), alkali treated and cellulose extracted AH filler. In the combination with cellulose AH filler varying from 0 to 5 wt%, 10–30 wt% NF (Fig. 5a), the better results were observed with 23.4 MPa for 20 wt% NF and 3 wt% cellulose filler combination. It is mainly due to presence of cellulose filler with –OH groups form better bonding with –OH group in 20 wt% NF creating better stress transferring capacity to polyethylene matrix^7,9^. It can be found that natural fibers with bamboo from 10 to 20 wt% showed a little improvement in the tensile strength (15–16.3 MPa) may be due to lesser interaction of lignocellulosic fibers with polymer composites. Cellulose filler with 3 wt% created better interaction with in the filler/fiber and with PE matrix, adding to the mechanical properties of polymer composites. An increase in 43.3, 43.6, 36.9% was observed in 10, 20 and 30 wt% NF combination with cellulose AH filler incorporation. The addition of cellulose filler at 5 wt% showed decline in the tensile strength due to agglomerated surface in cellulose filler making difficult to bond with NF fiber and PE matrix phase^10,32,33^. This cellulose filler addition method is an effective way for improving the mechanical application of natural fiber composites.

**Fig. 6:**
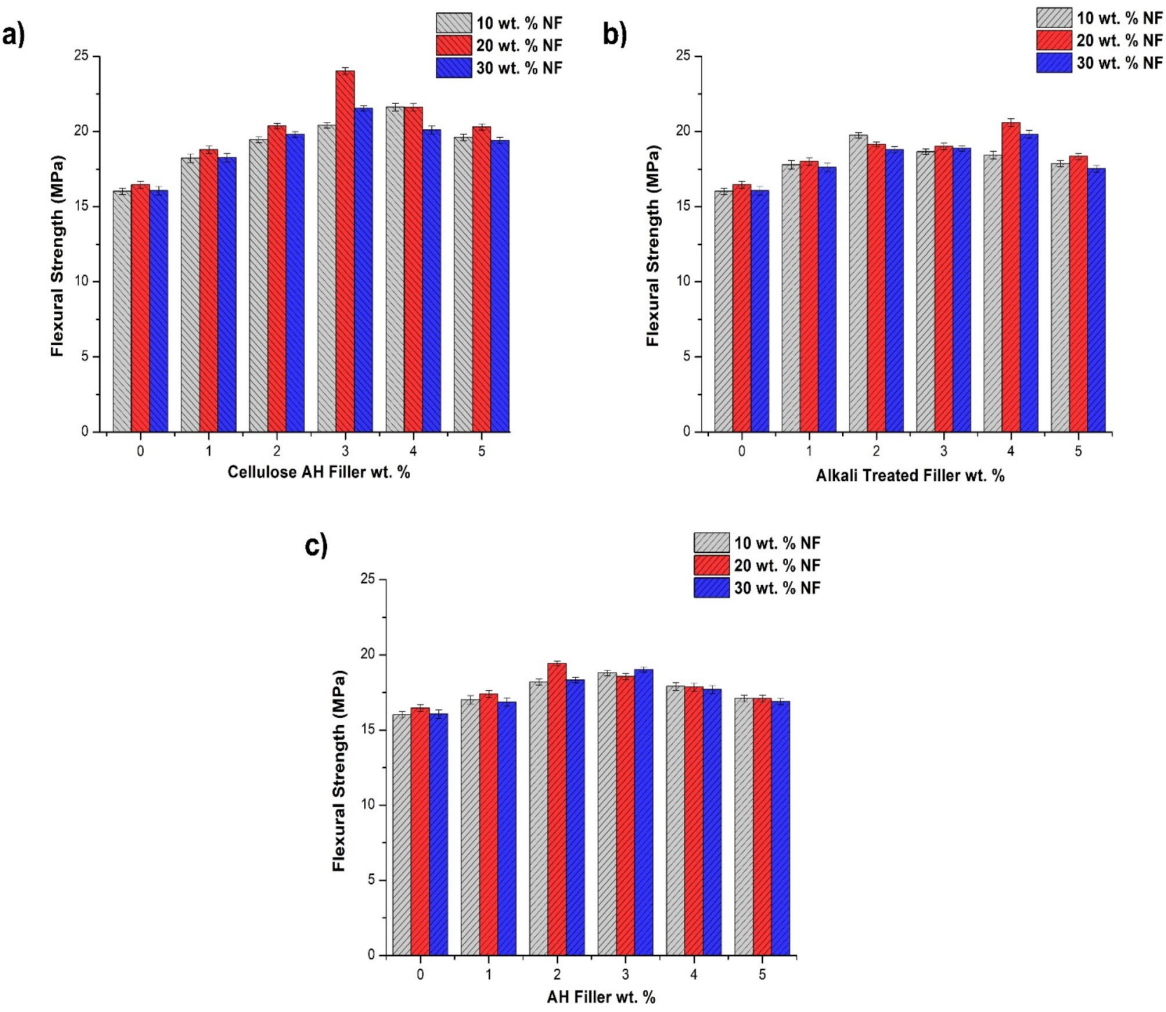
Flexural strength of bamboo fiber (NF)/*Artocarpus hirsutus* (AH) filler combinations (**a**) cellulose AH filler, (**b**) Alkali treated filler and (**c**) untreated AH filler.

The NaOH treatment in AH filler observed lesser tensile strength in comparison to cellulose based AH filler (Fig. 5b). The maximum properties were exhibited by 20 wt% NF/4 wt% filler with 19.6 MPa. In the NF combination of 10, 20 and 30 wt% an increase in 26.1, 20.2, 18.5% was observed for PE samples. Agglomeration declines the mechanical properties at higher filler incorporation in 4 wt% (10, 30 wt% NF), 5 wt% (20 wt% NF) alkali filler combination. The better results were observed in 20 wt% bamboo NF due to slightly even distribution and better bonding with fiber/filler/PE matrix phase^10,35^. In raw AH filler, the least improvement in tensile strength was observed (out of all other fillers) with 18.3 MPa at 20 wt% NF/2 wt% AH filler combination (Fig. 5c). This could be due to lower crystalline nature, cellulose content of the AH filler comparing to cellulose AH filler. Initial addition of filler at 1 wt% showed least impact in raw AH filler comparing to cellulose AH filler. The effectiveness in cellulose AH filler with 1 wt% incorporation observed 18.7% improvement in tensile properties at 10 wt% NF combination. Agglomeration at 4, 5 wt% AH filler addition declined the tensile properties, lesser than NF combination^11,36^. Tensile modulus increases with the addition of fillers and natural fibers (Fig. 6). The addition of natural fibers increases the tensile modulus from 434 to 524 MPa. Similarly, maximum increase in modulus from 555 to 649 MPa was observed with 30 wt% NF combination up to 5 wt% cellulose AH filler (Table 2). The alkali treated AH filler and untreated AH filler also observed the same trend with maximum tensile modulus results at 30 wt% NF/5 wt% filler incorporation^37,38^.

**Table 2:** Tensile modulus (TM) of bamboo fiber (NF)/*Artocarpus hirsutus* (AH) filler combinations.

	Cellulose AH	Cellulose AH	Cellulose AH	Alkali treated	Alkali treated	Alkali treated	AH filler	AH filler	AH filler
Filler Addition	10% NF	20% NF	30% NF	10% NF	20% NF	30% NF	10% NF	20% NF	30% NF
Unit	TM	TM	TM	TM	TM	TM	TM	TM	TM
wt%	MPa	MPa	MPa	MPa	MPa	MPa	MPa	MPa	MPa
0	434	484	524	434	484	524	434	484	524
1	453	492	555	441	497	565	431	495	545
2	480	515	576	470	510	579	460	504	569
3	493	535	600	497	538	610	487	518	582
4	515	576	629	518	578	632	500	538	608
5	540	621	649	535	624	640	520	564	621

### Flexural strength

The flexural strength showed similar results like tensile strength with 20 wt% bamboo NF combination having better flexural strength properties. The maximum flexural strength of 24.02 MPa was observed with a combination of 20 wt% NF/3 wt% cellulose AH filler (Fig. 6a). It can be seen from the older works that, natural fiber incorporation beyond the optimum level completely declines the flexural strength of polymer composites^11,32^. Cellulose filler created a smooth path with NF by better interaction with in fiber/fillers and with thermoplastic matrix, adding to the flexural strength of NF polyethylene combination^10,34^. Agglomeration at 4, 5 wt% affects the flexural properties by lesser interfacial adhesion with filler/matrix phase with properties reducing up to 20.3 MPa. Comparing to cellulose AH filler, both alkali treated and untreated AH filler posses lesser flexural strength (Fig. 6b,c), this could be due to richness in cellulose content comparing to hemicellulose, lignin adding to the strength.

The untreated, alkali treated AH filler showed maximum properties with 19.42, 20.59 MPa at 20 wt% NF combination. The alkali treated filler observed agglomeration at 5 wt% of filler incorporation and untreated filler with 4, 5 wt% filler addition at PE matrix. The Flexural modulus observed betterment with all the filler addition and also with bamboo fiber incorporation (Table 3). Natural fiber showed 718 MPa in flexural modulus results at 30 wt% addition with polyethylene matrix. The hybridization of filler with NF effect enhances the modulus results from 718 to 816 MPa at 5 wt% filler substitution. The maximum modulus results of 804 MPa, 810 MPa was observed with untreated filler, alkali treated filler with 30 wt% natural fiber combination^39,40^.

**Table 3:** Flexural modulus (FM) of bamboo fiber (NF)/*Artocarpus hirsutus* (AH) filler Combinations.

	Cellulose AH	Cellulose AH	Cellulose AH	Alkali treated	Alkali treated	Alkali treated	AH filler	AH filler	AH filler
Filler Addition	10% NF	20% NF	30% NF	10% NF	20% NF	30% NF	10% NF	20% NF	30% NF
Unit	FM	FM	FM	FM	FM	FM	FM	FM	FM
wt%	MPa	MPa	MPa	MPa	MPa	MPa	MPa	MPa	MPa
0	674	692	718	674	692	718	674	692	718
1	683	712	724	680	702	726	682	705	729
2	710	734	745	705	721	746	708	726	748
3	745	750	768	738	745	763	743	746	765
4	762	772	793	752	762	784	750	758	782
5	784	796	816	775	784	810	771	781	804

### Impact testing and water absorption properties

**Fig. 7:**
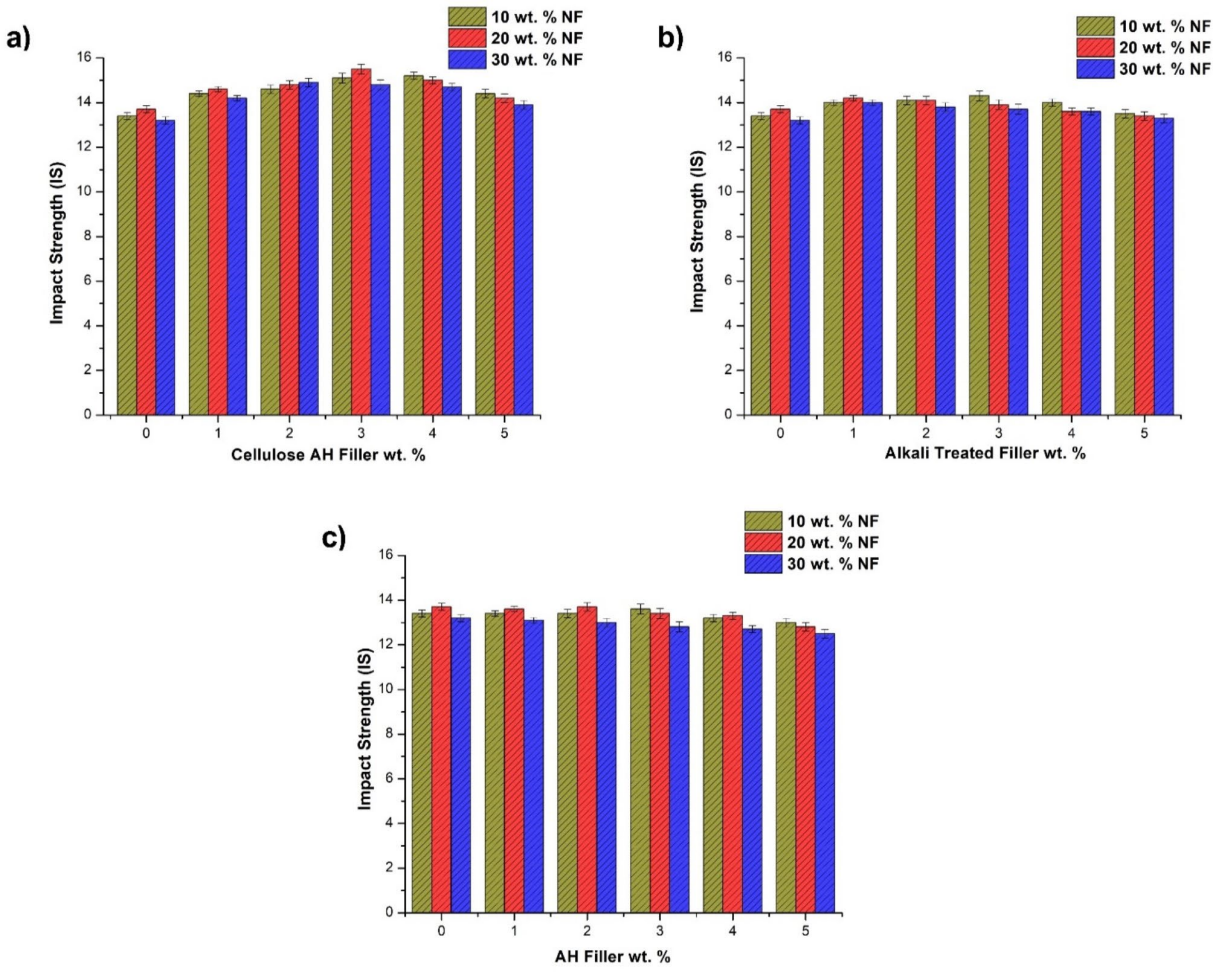
Impact strength of bamboo fiber (NF)/*Artocarpus hirsutus* (AH) filler combinations (**a**) cellulose AH filler, (**b**) Alkali treated filler and (**c**) untreated AH filler.

**Fig. 8:**
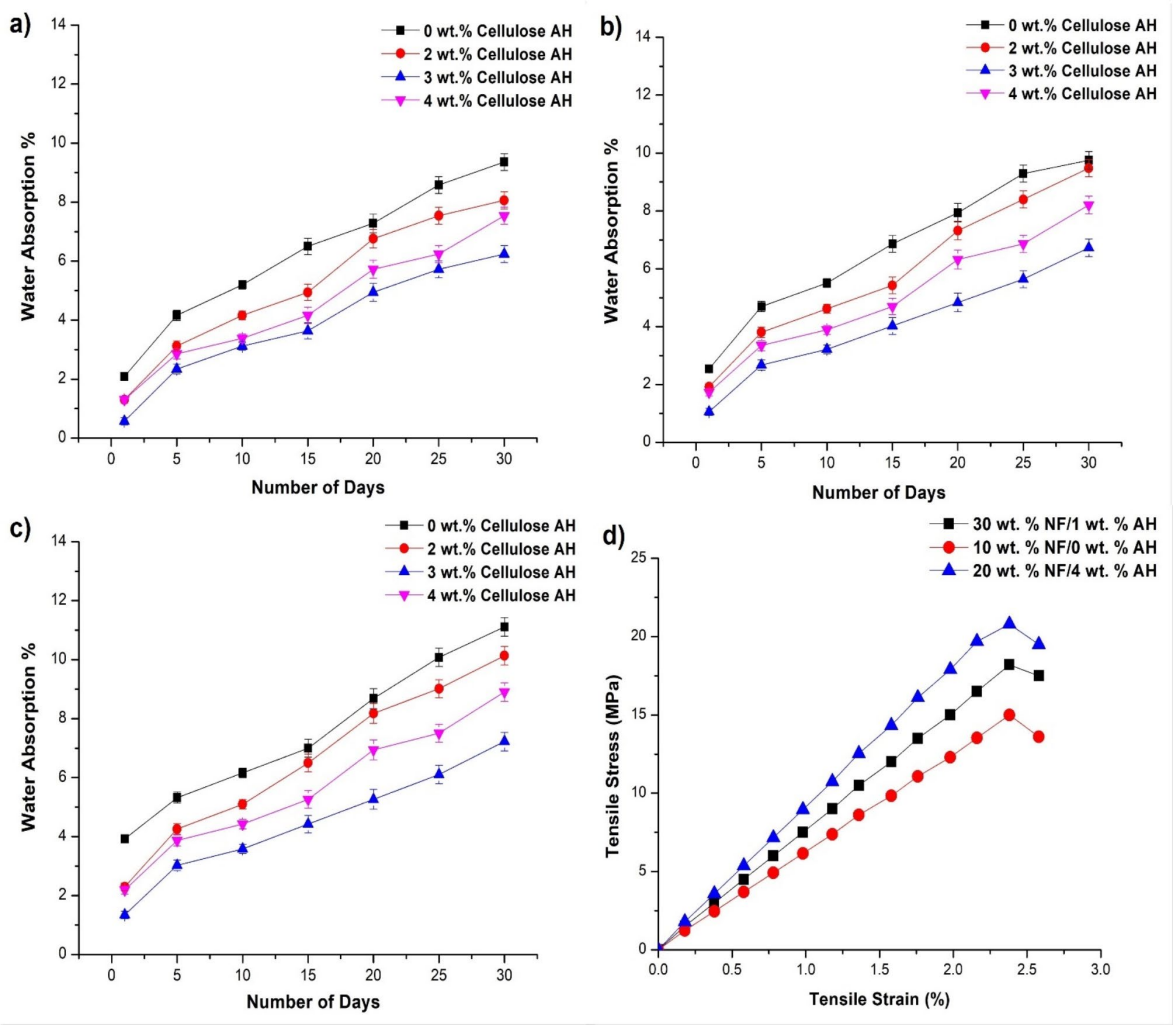
Water absorption percentage of (**a**) 10 wt% NF, (**b**) 20 wt% NF, (**c**) 30 wt% NF with cellulose AH Filler, (**d**) Tensile Stress–Strain Graph.

The Impact strength doesn’t observe high influence with filler incorporation, comparing to both tensile and flexural strength (Fig. 7). The incorporation of bamboo fiber showed a little improvement in the impact strength (13.4–13.7 kJ/m^2^) (Fig. 7a). The cellulose filler incorporation enhances the impact results from 13.7 to 15.5 kJ/m^2^ in 20 wt% NF at 3 wt% filler addition. Various treatments with filler contributed to its crystallinity, that leads to enhancement in impact strength of cellulose filler composites. The influence of alkali treated AH filler (Fig. 7b), untreated fillers with PE matrix (Fig. 7c) for impact strength is lesser in comparison to cellulose based AH filler. The alkali treated filler slightly improved the impact properties (13–7–14.1 kJ/m^2^) of natural fiber PE composites^41–43^. The untreated filler declines the properties of NF/PE based composites. Water absorption properties observed increase in water absorption with the bamboo NF incorporation up to 30 wt% (Fig. 8). It was evident that natural fibers with hydrophilic nature increases the water absorption nature of polymer composites^9,37^. In the case of cellulose AH filler, least water absorption rate was observed with 3 wt% Cellulose AH filler, it may be due to the reduction of void space between fiber/matrix with filler incorporation^11,43^. This filler replaces the gaps of the interface and reducing the water absorption rate. Tensile stress strain graph of three hybrid combinations were observed in Fig. 8d.

### Void percentage, hardness and density

**Fig. 9:**
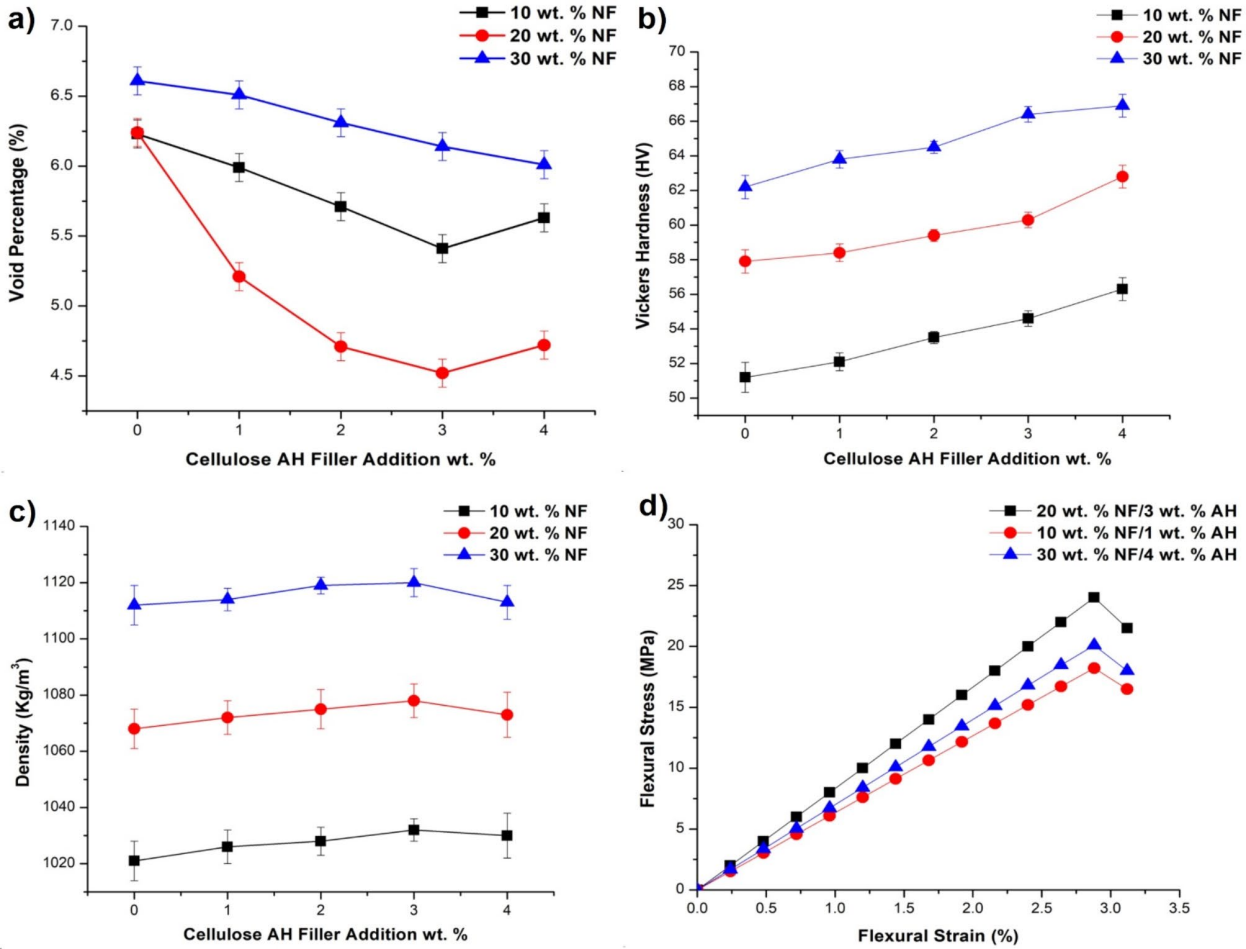
Cellulose AH filler composites. (**a**) void percentage, (**b**) Hardness, (**c**) Density, (**d**) flexural stress strain graph.

In the void percentage 20 wt% bamboo NF combinations has the least voids due to presence of cellulose AH filler clearing the void gaps (Fig. 9a). All the 10–30 wt% NF combination had lesser void space until 3 wt% of filler incorporation. Agglomeration at 4 wt% reduces the properties beyond that^36^. Hardness property increases mainly with the natural fiber incorporation than filler addition (Fig. 9b). Maximum increase was observed at 30 wt% NF/4 wt% Cellulose AH filler combination with 65 HV (Fig. 9c). Density property is also similar to hardness with increase in property predominantly due to natural fiber incorporation (Fig. 9d)^38^.

### Thermal results

**Fig. 10:**
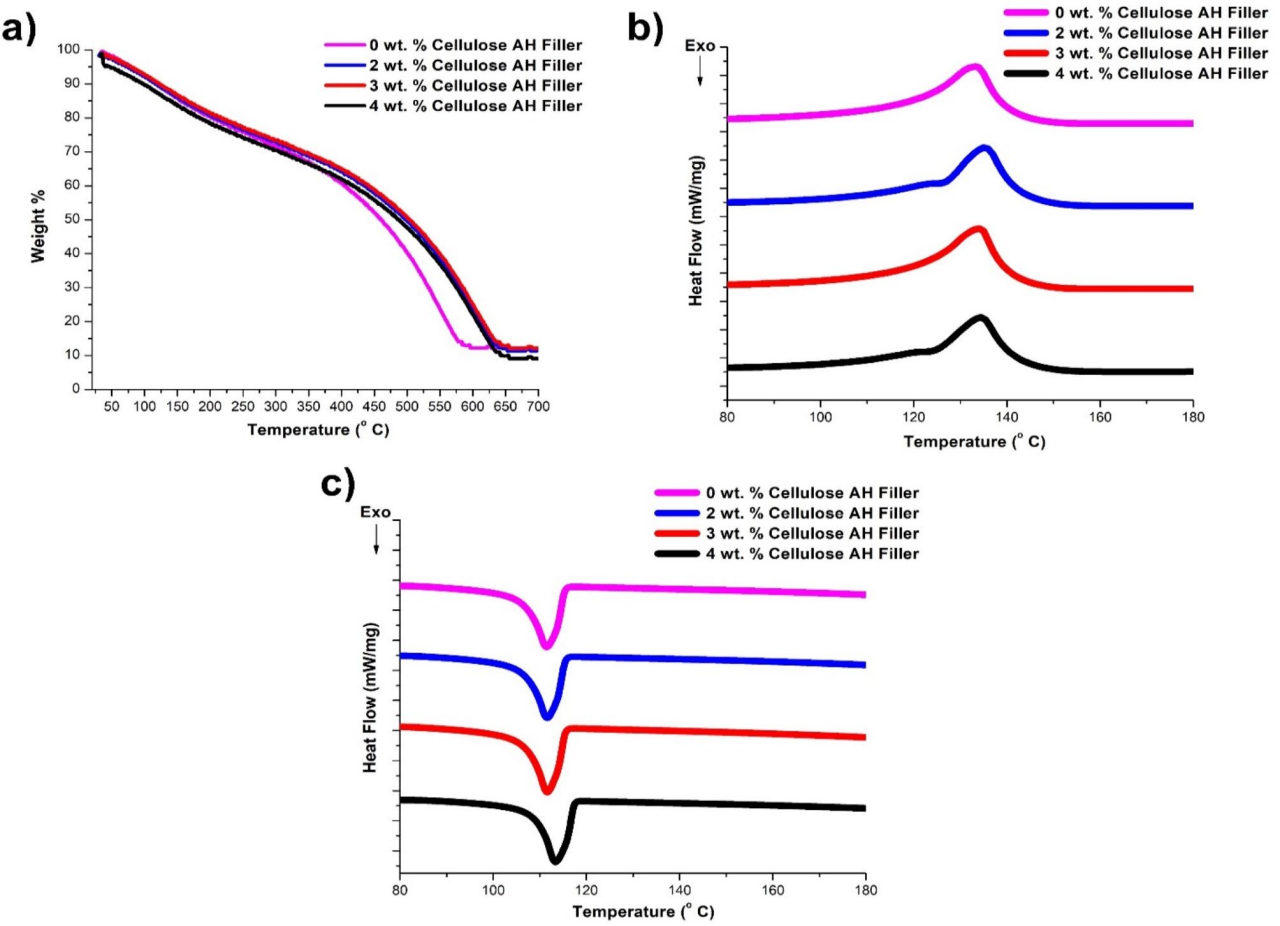
(**a**) TGA and (**b**), (**c**) DSC results of 20 wt% bamboo fiber (NF)/Cellulose *Artocarpus hirsutus* (AH) filler combinations.

The degradation at 10, 50, 70% along with residual weight percentage determines the thermal stability of the hybrid polymer composites (Fig. 10a). The initial degradation at 10% was observed at 115–117 °C for 0–4 wt% of cellulose AH filler incorporation with 20 wt% NF, this degradation could be due to evaporation of liquids from bio composite samples. The secondary degradation at 50% was observed at 498 °C, 503 °C in 2, 3 wt% cellulose filler incorporation comparing to 1, 4 wt% filler with 461 °C, 488 °C. The third or final type of degradation of 70% at 580 °C (1 wt% filler), 584 °C (2 wt% filler) and 531 °C (0 wt% filler), 575 °C (4 wt% filler). The residual weight percentage of 11.8–12.1% was observed from 0 to 3 wt% filler incorporation. The DSC results with melting temperature (132.2–133.1 °C) (Fig. 10b), crystallization temperature (111.2–113.4 °C) (Fig. 10c) doesn’t show any changes with the incorporation of cellulose filler addition. The enthalpy of heat at melting showed slight improvement from 114.2 to 116.4 J/g with 0–4 wt% filler addition. Similarly, enthalpy of crystallization observed increase from − 104.3 to − 96.6 J/g (0–4 wt% filler addition)^41,44^.

### SEM results

**Fig. 11:**
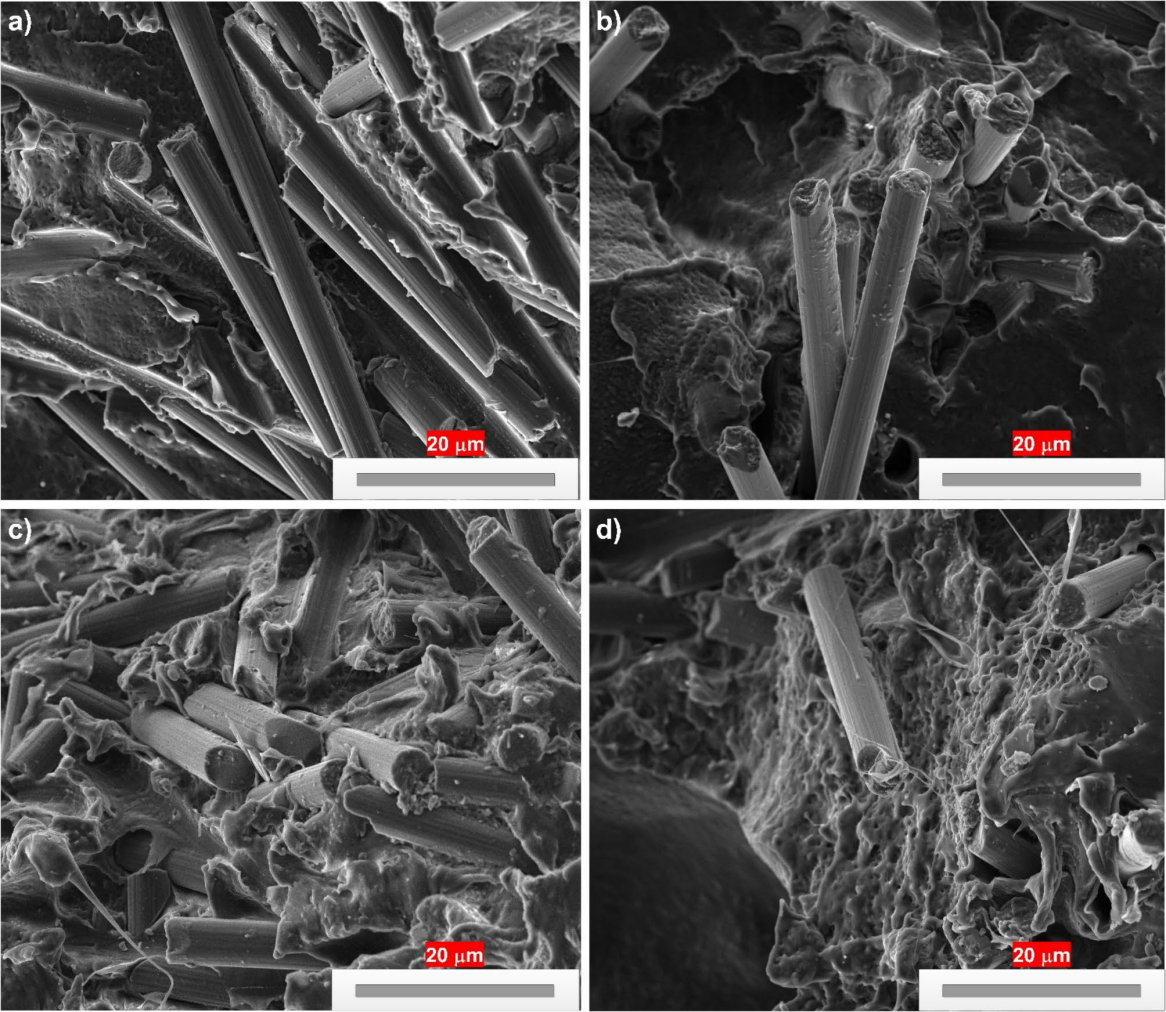
SEM Results of 20 wt% NF/PE combinations at (**a**) 0 wt% Cellulose AH filler, (**b**) 2 wt% Cellulose AH filler, (**c**) 3 wt% Cellulose AH filler, (**d**) 5 wt% Cellulose AH filler at 20 microns.

The combination with 0 wt% Cellulose AH filler/20 wt% NF observed clear gap between matrix/fiber, matrix crack, fiber debonding leading to sample with poor mechanical properties (Fig. 11a). The substitution of filler at 2 wt% slightly (Fig. 11b) improved the structure with better interfacial adhesion with fillers and matrix. Filler addition at 3 wt% created a better bonding with filler/fiber/matrix, improving stress transfer capacity of PE matrix (Fig. 11c). The morphological analysis of hybrid combinations observed better interaction with bamboo NF/PE matrix and with the incorporation of cellulose AH filler. The agglomeration at 5 wt% cellulose filler (Fig. 11d) created more surface deformations in PE matrix leading to lesser mechanical properties.

## Conclusions

- The FTIR results observed presence of a peak at 1101 cm^−1^ indicated the stretching vibration of a C-O group in the untreated AH filler material. Specific vibrational modes of cellulose molecules, with the peak at 1061 cm^−1^ typically associated with the stretching vibration of C–O–C bonds, and the peak at 1163 cm^−1^ associated with stretching vibration of C-O bonds in cellulose.
- The 10, 20 and 30 wt% bamboo natural fibers (NF) were incorporated with 0–5 wt% of *AH * filler, alkali treated filler and cellulose extracted AH biomaterial filler. In the combination with cellulose AH filler varying from 0 to 5 wt%/10 to 30 wt% NF, the better results were observed with 23.4 MPa for 20 wt% NF and 3 wt% cellulose filler biocomposites. It is mainly due to presence of cellulose filler with –OH groups form better bonding with –OH group in 20 wt% NF creating better stress transferring capacity to polyethylene matrix.
- The addition of natural fibers increases the tensile modulus from 434 to 524 MPa. Similarly, maximum increase in modulus from 555 to 649 MPa was observed with 30 wt% NF combination up to 5 wt% cellulose AH filler. The alkali treated AH filler and untreated AH filler also observed the same trend with maximum tensile modulus results at 30 wt% NF/5 wt% filler incorporation. Enhancing the biocomposite applications.
- The maximum flexural strength of 24.02 MPa was observed with a combination of 20 wt% NF/3 wt% cellulose AH filler. Cellulose filler created a smooth path with NF by better interaction with in fiber/fillers and with thermoplastic matrix, adding to the flexural strength of NF polyethylene combination. Agglomeration at 4, 5 wt% affects the flexural properties by lesser interfacial adhesion with filler/matrix phase thus properties reduced up to 20.3 MPa.
- The Impact strength doesn’t observe high influence with filler incorporation comparing to both tensile and flexural strength. The cellulose filler incorporation enhances the impact results from 13.7 to 15.5 kJ/m^2^ in 20 wt% NF at 3 wt% filler addition. The influence of alkali treated AH filler, untreated fillers with PE matrix for impact strength is lesser in comparison to cellulose based AH filler.
- The combination with 0 wt% Cellulose AH filler/30 wt% NF observed clear gap between matrix/fiber, matrix crack, fiber debonding in SEM analysis. Filler addition at 3 wt% created a better bonding with filler/fiber/matrix, improving stress transfer capacity of PE matrix. The morphological analysis of hybrid combinations observed better interfacial adhesion with bamboo and PE matrix by incorporation of cellulose AH filler.
- This cellulose filler addition method is an effective way for improving the mechanical application of biocomposites and this fabricated composite is suited for bioengineering applications.

## Data Availability

The necessary data used in the manuscript are already present in the manuscript.
